# Drinking practices: The variation of drinking events across intersections of sex, age and household income

**DOI:** 10.1111/dar.13975

**Published:** 2024-11-13

**Authors:** Petra S. Meier, John Holmes, Abigail Stevely, Jennifer E. Boyd, Monica Hernández Alava, Iain Hardie, Alan Warde, Alessandro Sasso

**Affiliations:** ^1^ MRC/CSO Social and Public Health Sciences Unit, School of Health and Wellbeing University of Glasgow Glasgow UK; ^2^ Sheffield Addictions Research Group, Sheffield Centre for Health and Related Research University of Sheffield Sheffield UK; ^3^ Salvation Army Centre for Addiction Services and Research University of Stirling Stirling UK; ^4^ The University of Edinburgh School of Philosophy Psychology and Language Sciences Edinburgh UK; ^5^ Sustainable Consumption Institute University of Manchester Manchester UK; ^6^ European Commission Joint Research Center Ispra Italy

**Keywords:** alcohol drinking, health inequities, lifecourse, social practices, socioeconomic factors

## Abstract

**Introduction:**

Investigations of drinking practices often rely on cross‐country comparisons of population averages in beverage preferences, drinking volumes and frequencies. Here, we investigate within‐culture patterns and variations in where, why and how people drink, answering the research question: how does engagement in drinking practices vary by sex, age and household income?

**Methods:**

We conducted a cross‐sectional analysis examining the societal distribution (by age, sex, household income) of 12 drinking practices: four off‐trade practices (in‐home consumption; e.g., evening at home with partner) and eight on‐trade practices (licensed‐venue consumption, e.g., family meal, big night out). Practices were identified in previous analyses of 2019 British event‐level diary data (14,742 drinkers aged 18+ reporting 26,220 off‐trade and 8768 on‐trade occasions).

**Results:**

The level of engagement in practices varied by sex, age and income. In the on‐trade sector, men, particularly those in low‐income groups, engaged in traditional pub‐drinking, while women, especially older women, engaged in sociable drinking occasions with family and friends which commonly involved food. Young men and women were similarly likely to engage in heavier on‐trade practices, which remained commonplace into midlife. Drinking while socialising with friends, both inside and outside the home, was common among younger age groups across all income bands. From midlife, home drinking often involved a partner, especially for higher income groups.

**Discussion and Conclusions:**

Most drinking practices were shared across the whole population, but level of engagement in them is strongly patterned by age, household income and, particularly in the on‐trade sector, sex.

**Key Points**

- There is a lack of quantitative evidence on within‐culture variability in the drinking habits of different subgroups of the population.
- British drinking diary data was used to investigate the degree to which societal groups defined by age, sex and income engage in shared or separate drinking practices.
- Outside the home, women's practices focused on lighter, meal‐based drinking and drinking with a partner, particularly at older ages. Conversely, men engaged in more “traditional” pub drinking, particularly among lower income groups.
- In the home, where the majority of British drinking occurs, drinking practices appeared bound up with age and household type. Socialising‐focused drinking with friends, both inside and outside the home, was common among younger age groups across all incomes. For older, low‐income men, quiet drinks at home, often alone, accounted for over half of drinking occasions, while among men and women with higher incomes, drinking with a partner or family became increasingly prominent with age.

## 1 INTRODUCTION

A dominant quantitative approach to studying national drinking cultures is to characterise countries using a ‘dimensional approach’, which focuses on one or few dimensions of drinking only (e.g., wet versus dry cultures, regularity or functionality of drinking) [1, 2]. This approach, while useful, provides limited insight into important within‐country variation in drinking behaviour between drinker subgroups [3, 4]. Consequently, to characterise and study variations of within‐country drinking cultures, we previously proposed an alternative approach informed by social practice theory [5, 6], identified suitable data, and used this to examine stability and change in the engagement with different drinking practices over time [7] and examine the types of drinking practices most associated with heavy episodic drinking [8].

Many versions of practice theory exist [9] but most focus on understandings and arrangements for commonly occurring events, considering the coming together of people, contexts, settings, activities and sequences into routines widely recognisable by members of the same cultural background (e.g., glass of wine with food at a restaurant, beer with TV sports). The theory further posits that participants are attuned to the norms of the practice, responsive to the cues transmitted by the structuring of the situation, and have an inclination to conform to the shared and common purposes of relevant others [10, 11].

Within‐country variations in drinking behaviour by gender, age and socio‐economic position (SEP) have most commonly been explored quantitatively by taking an epidemiological rather than drinking practices approach, especially examining variations in the frequency or volume of alcohol consumption [12, 13, 14]. For Britain, such research has highlighted important age‐sex trends. For example, until recently, both men and women drank most heavily in their mid‐20s [15, 16]. However, recent declines in youth drinking mean consumption now peaks in middle‐age, with men continuing to drink more frequently, and in greater quantities, than women [17]. At the same time, the relationship between alcohol consumption and SEP remains complex. The ‘alcohol harm paradox’, refers to consistent evidence that lower SEP groups experience disproportionately high rates of alcohol‐related harm despite drinking less [18, 19, 20]. The paradox has been partially explained by differences in drinking practices, for example, findings that distinct cultural norms may govern how alcohol is consumed by different groups or that occasions in certain settings are associated with higher risk of violence, injuries and police encounters [21, 22, 23, 24].

However, only few studies researching drinking practices have adopted an intersectional lens (i.e., examining how drinking varies at the intersections between sex/age/SEP, e.g., affluent young women or low‐income elderly). A notable exception is Whitley et al. [25] who were able to show that male and female manual workers were more likely to drink heavily than non‐manual workers at older ages, but not younger ages. Qualitative research provides more detailed insights, albeit focused on specific drinking practices or subgroups, for example, the role of drinking practices in male friendships, or demarcating roles such as mother and partner [26, 27]. However, we still have little systematic quantitative understanding about which population groups engage in which practices. This paper aims to carry out an intersectional analysis of drinking practices and answer the research question ‘To what extent are drinking practices shared by men and women of different ages and income levels?’

## 2 METHODS

### 2.1 Data and sample

We used 2019 Kantar Alcovision data, a repeat cross‐sectional online market research survey of 16,147 adult drinkers in Great Britain and detailed, 1‐week, retrospective drinking diary of respondents' drinking occasions. Given a non‐probabilistic sample design of Kantar Alcovision (quota sampling) and the deliberate oversampling of 18–24 year olds and individuals in Scotland, we used an iterative proportional fitting, or raking, technique to calibrate survey weights to the UK Census population (full detail in the online appendix to [8]). Of 16,145 respondents reporting one or more drinking occasions in the diary week, 1403 (8.7%) were excluded due to missing income data, giving a final sample of *n* = 14,742 adults reporting 8768 on‐trade and 26,220 off‐trade occasions.

### 2.2 Measures

Respondents provided information on sex, age (in years) and gross annual household income (including wages, benefits, pension, investments; five approximately equal‐sized bands <£10,000; £10,000–£19,999; £20,000–£34,999; £35,000–£54,999; £55,000+). Respondents were asked to report details of their past‐week drinking occasions, including the following occasion characteristics: (i) type of venue (e.g., own home/pub/restaurant) and, for on‐trade occasions, reasons for venue choice (e.g., cheap/convenient/food quality) and location (e.g., city centre/entertainment complex/rural); (ii) group composition (e.g., mixed‐sex friend group/family members); (iii) occasion timing (weekday/time/duration); (iv) occasion purpose (e.g., family occasion/winding down); (v) food consumption (e.g., no food/snack/full meal); (vi) activities involved (e.g., TV watching/pub quiz/live music); and (vii) alcohol consumed, that is, beverage type and occasion‐level consumption.

### 2.3 Conversion of drinking occasions into practices

We draw on a drinking practice typology developed in previous work [7, 8] where four off‐trade (drinking shop‐bought alcohol in one's own home/someone else's home/park etc) and eight on‐trade (drinking in licensed premises, e.g., pubs/bars/restaurants/nightclubs) drinking practices were identified through latent class analysis. In prior work, mixed on‐/off‐trade practices were also described but are excluded here as they represent a minority of drinking occasions and breakdown by age × sex × income subgroup was not appropriate. Table 1 shows characteristics of each drinking practice, and provides information on mean consumption and the proportion of occasions in each practice that are ‘heavy drinking occasions’, defined as >6 units (48 g ethanol) for women and >8 units (64 g ethanol) for men.

**TABLE 1:** Drinking practice typology for Great Britain, 2019: off‐trade (blue shading) and on‐trade (white) drinking practices, with mean consumption and % that are heavy drinking occasions (adapted from reference [8]).

Off‐trade practices			
Quiet drink at home	Family time at home	Evening at home with partner	Off‐trade get together
28.5% of off‐trade occasions	13.5% of off‐trade occasions	34.1% of off‐trade occasions	23.9% of off‐trade occasions
Mean (SD) units: 6.2 (6.7)	Mean (SD) units: 6.9 (7.0)	Mean (SD) units: 5.9 (5.8)	Mean (SD) units: 10.4 (10.0)
27.3% are HDOs^a^	32.3% are HDOs	27.4% are HDOs	48.1% are HDOs
Nearly always^b^: Own home, less than 4 h.	Nearly always: Mixed sex group, partner or family, own home, less than 4 h.	Nearly always: Mixed sex pair, partner, own home, less than 4 h.	Nearly always: N/A
Commonly: Alone, quiet or regular drink, watching TV, no food, evening, Monday–Friday, Friday–Saturday.	Commonly: Quiet or regular drink, watching TV, meal, Friday–Saturday.	Commonly: Quiet or regular drink, watching TV, meal, evening, Friday–Saturday, wine.	Commonly: Mixed sex group, friends, own or other's home, sociable, games or leisure activities, meal, less than 4 h, evening, Friday–Saturday.

On‐trade practices			
Meeting friends at the pub	Male friends at the pub	Quiet drink at the pub	Family meal
18.0% of on‐trade occasions	12.9% of on‐trade occasions	13.8% of on‐trade occasions	11.2% of on‐trade occasions
Mean (SD) units: 7.4 (6.1)	Mean (SD) units: 10.2 (6.7)	Mean (SD) units: 5.5 (4.6)	Mean (SD) units: 4.4 (4.3)
40.4% are HDOs	55.7% are HDOs	23.5% are HDOs	17.0% are HDOs
Nearly always: Mixed sex group.	Nearly always: Friends, male group or pair.	Nearly always: N/A	Nearly always: N/A
Commonly: Friends, traditional pub, regular or local place, no food, less than 4 h, evening, Friday–Saturday, beer.	Commonly: Traditional pub, regular or local place, convenient location, no food, evening, 1–4 h, Friday–Saturday, beer.	Commonly: Male alone, traditional pub, convenient place, no food, Monday–Friday, beer.	Commonly: Mixed sex group, family, food pub or restaurant, quality of food or drinks, meal, lunchtime or afternoon, beer.
**Meal with friends**	**Going out with the partner**	**Big night out**	**Extended occasion**
10.8% of on‐trade occasions	12.7% of on‐trade occasions	6.1% of on‐trade occasions	14.4% of on‐trade occasions
Mean (SD) units: 5.3 (5.3)	Mean (SD) units: 5.1 (4.8)	Mean (SD) units: 11.1 (9.1)	Mean (SD) units: 15.9 (12.2)
24.1% are HDOs	22.5% are HDOs	58.3% are HDOs	68.9% are HDOs
Nearly always: N/A	Nearly always: Mixed sex pair, partner, less than 4 h.	Nearly always: Evening or night‐time.	Nearly always: N/A
Commonly: Mixed sex group, friends, food pub or restaurant, quality of food or drinks, meal, 1–4 h, Monday–Friday, Friday–Saturday.	Commonly: Food pub or restaurant, quality of food or drinks, convenient location, having time for partner, meal, no food, evening, Friday–Saturday, beer.	Commonly: Mixed sex group, friends, club, city centre, clubbing or night out, having a laugh, live music, no food, 1–4 h, Friday–Saturday, spirits.	Commonly: Mixed sex group, friends, child present, multiple venues, traditional pub, quality of food and drinks, games, meal, 1–4 h, more than 4 h, evening, Monday–Friday, Friday–Saturday.

Abbreviation: N/A, not applicable.

^a^ HDO, heavy drinking occasion, defined as >6 units (48 g ethanol) for women and >8 units (64 g ethanol) for men.

^b^ ‘Nearly always’: item response probability ≥0.9. ‘Commonly’: item response probability is ≥0.4 and <0.9.

### 2.4 Relationship between drinking practices and individual characteristics

This paper uses previously estimated drinking practices as inputs. We analyse the relationship between type of occasions (an occasion‐level variable) and sex, age and income (individual‐level variables). We do this by estimating the probability that an occasion falls within a given class in the latent class model (i.e., it is a performance of a particular practice) as a function of respondent sex/age/income. The analysis is at the occasion level, but occasions cluster within individuals. Therefore, we clustered standard errors at the individual level to account for the fact that the model errors may be correlated within a cluster. Details of the latent class analyses conducted to estimate drinking practices are provided in Holmes et al. [8] but, in brief, we used Vermunt's bias‐adjusted three‐step procedure [28]: latent class estimation to identify drinking practices; probabilistic assignment of each drinking occasion in the dataset to one of the drinking practices; and estimation of the relationship between drinking practices and individual‐level characteristics (age, sex and income; such variables are sometimes called auxiliary variables in latent class analyses). The model specification was multinomial logistic regression, a commonly used model to analyse the relationship between a categorical dependent variable (in this case, the type of occasion) and a set of explanatory variables or predictors (in this case, gender, age, age squared and income). Specifically, the multinomial logit model is described by the following equation:

(1) $$P\left({\text{occasion}}_{i}=k|x_{i}\right)=\frac{\exp\left(x_{i}\delta_{k}\right)}{\sum_{j=1}^{C}\exp\left(x_{i}\delta_{j}\right)}$$

where occasion is a categorical variable in the range *k* = (0, 1,…, C) referring to the type of occasion observed for an observation i; 𝑥 includes the set of predictors (gender, age, age squared and income); 𝛿 denotes the parameter estimate describing the relationship between the predictor of interest and the probability that an occasion falls within a particular latent class. Analyses were performed using MPLUS8 [29].

### 2.5 Visualisation of results

In the online appendix, we provide information on the number of occasions reported by each subgroup (Table S1) and multinomial logistic regression results (Tables S2 and S3). Here, our focus is not on the differences between any two specific subgroups but to show overarching intersectional patterns and so visualisations and description of results attempt to make transparent some of the key sex‐by‐age‐by‐income patterns in the engagement across the 12 drinking practices.

**FIGURE 1:**
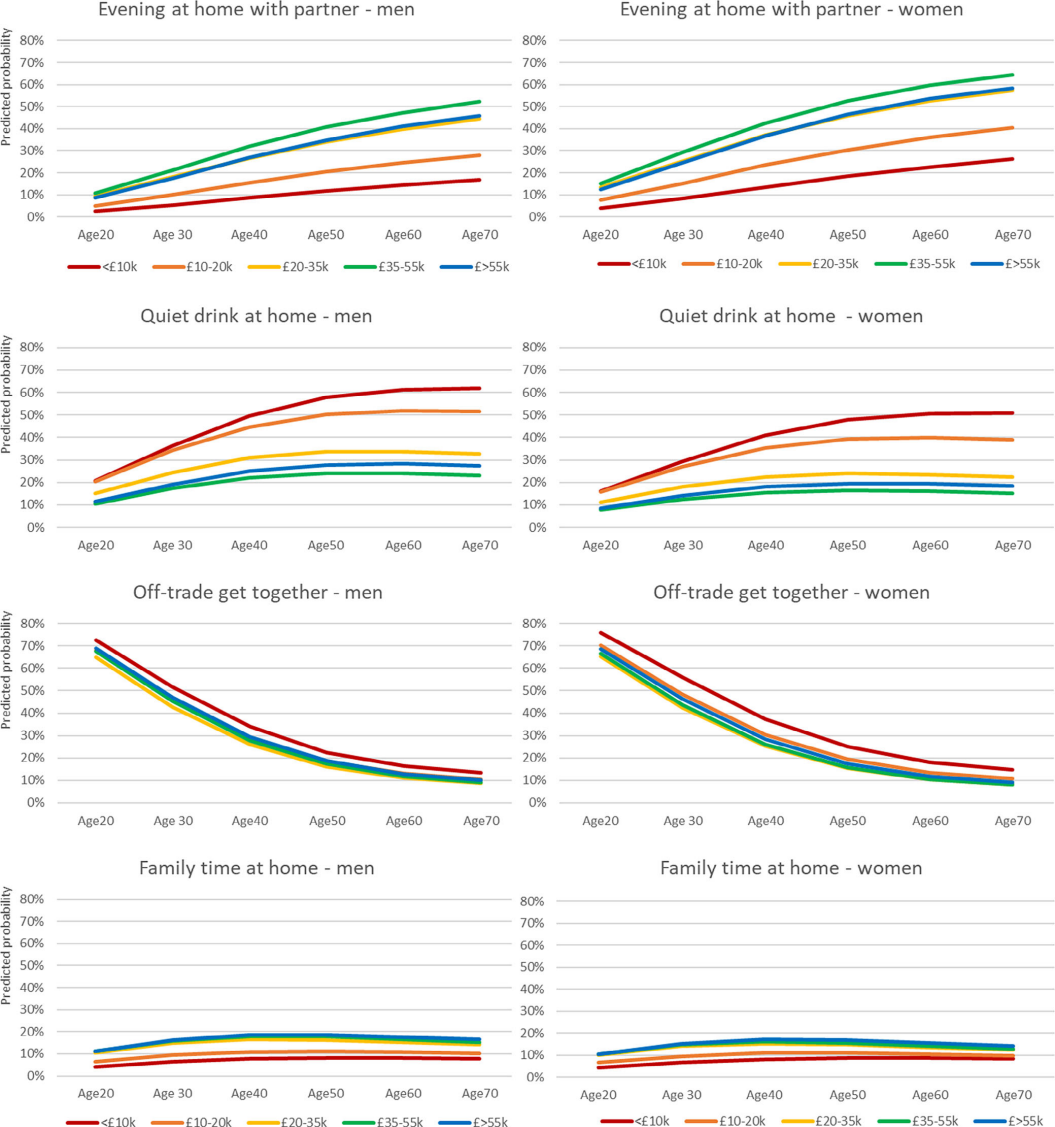
Off‐trade drinking practices: For each age (x‐axis), sex (column of graphs) and income group (coloured lines on graphs), the proportion of their off‐trade occasions that are performances of each practice.

**FIGURE 2:**
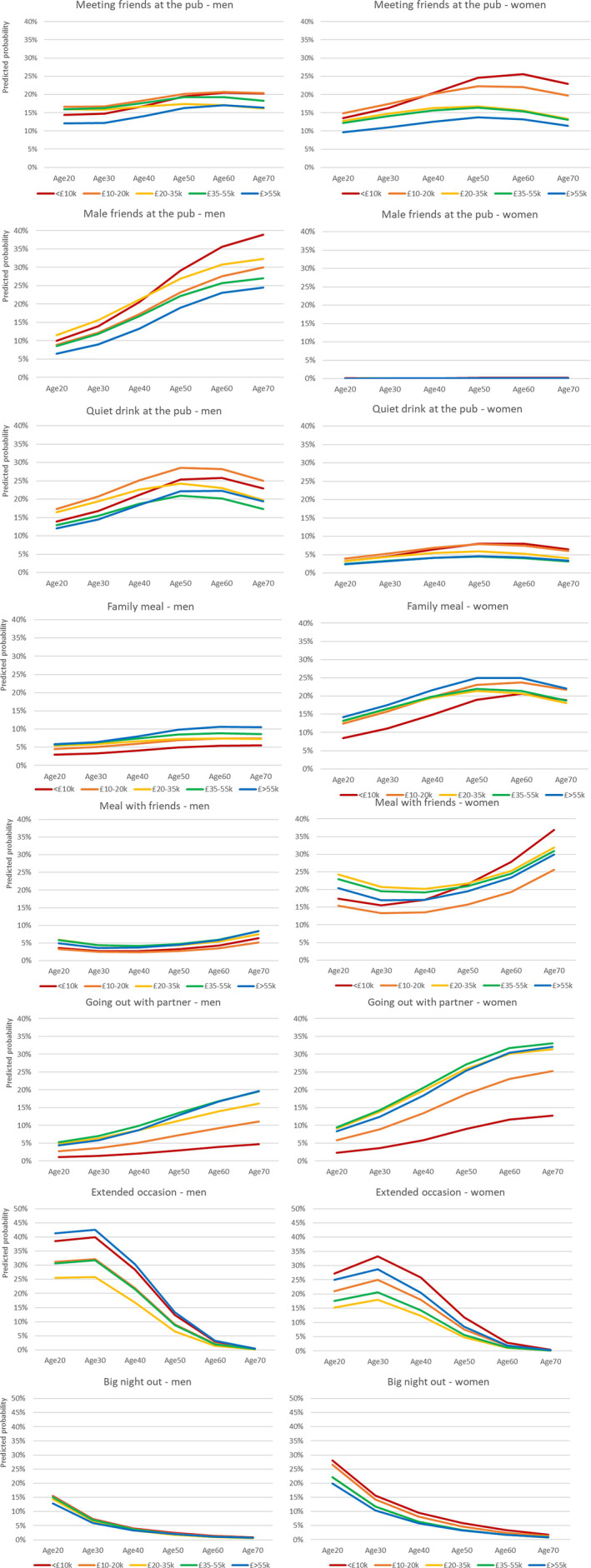
On‐trade drinking practices: For each age (x‐axis), sex (column of graphs) and income group (coloured lines on graphs), the predicted proportion of their on‐trade occasions that are performances of each practice.

Figures 1, 2 and Tables 2, 3 were designed to visualise the share of occasions accounted for by each off‐trade and on‐trade drinking practice, respectively, given respondents' sex, age and income. The graphs were designed to facilitate different visual comparisons:

1. Age: How does the probability of an occasion being a performance of a given practice vary by age (horizontal comparison within same graph).
2. Income: How does the probability of an occasion being a performance of a given practice vary by income group? (vertical comparison of different‐coloured lines within same graph).
3. Sex: How does the probability of an occasion being a performance of a given practice vary by sex? (horizontal comparison of same‐coloured lines across adjacent graphs).
4. How does each age‐sex‐income group distribute its drinking occasions across different drinking practices? (vertical comparison of lines of the same colour, and points on those lines, for graphs in same column).

**TABLE 2:** Off‐trade heatmap: For each given age‐sex‐income group, the predicted proportion of their off‐trade occasions that are performances of each practice.

Income ↓		Men						Women					
Age →		20	30	40	50	60	70	20	30	40	50	60	70
Off‐trade practices		%	%	%	%	%	%	%	%	%	%	%	%
Evening at home with partner	<£10,000 k	2.6	5.4	8.6	11.6	14.4	16.8	3.8	8.2	13.5	18.4	22.6	26.2
	£10,000–19,999 k	5.1	10.2	15.6	20.4	24.4	28.0	7.5	15.2	23.4	30.4	36.0	40.5
	£20,000–34,999 k	9.3	18.0	26.7	34.0	39.8	44.4	13.3	25.4	37.0	46	52.6	57.5
	£35,000–54,999 k	10.8	21.3	32.0	40.7	47.3	52.3	15.2	29.2	42.6	52.6	59.6	64.5
	£55,000 k+	8.8	17.6	26.9	34.8	41.1	45.9	12.4	24.6	36.8	46.4	53.5	58.5
Quiet drink at home	<£10,000 k	20.6	36.4	49.7	57.7	61.2	62	16.0	29.3	40.9	47.9	50.7	50.8
	£10,000–19,999 k	20.4	34.3	44.7	50.1	51.8	51.4	15.7	26.9	35.3	39.2	40	39
	£20,000–34,999 k	15.0	24.4	30.9	33.6	33.8	32.7	11.2	18.1	22.4	23.9	23.5	22.3
	£35,000–54,999 k	10.4	17.4	22.2	24.2	24.2	23.2	7.7	12.5	15.5	16.4	16.0	15.0
	£55,000 k+	11.3	19.2	25.1	27.8	28.2	27.4	8.4	14.1	18.0	19.4	19.3	18.3
Off‐trade get together	<£10,000 k	72.6	51.8	33.9	22.5	16.3	13.4	76.0	55.9	37.5	25.1	18.1	14.8
	£10,000–19,999 k	67.9	46.0	28.8	18.5	13.0	10.5	70.3	48.4	30.4	19.4	13.5	10.7
	£20,000–34,999 k	65.1	42.8	25.9	16.2	11.1	8.7	65.4	42.5	25.3	15.4	10.4	8.0
	£35,000–54,999 k	67.5	45.3	27.8	17.3	11.8	9.2	66.7	43.7	26.0	15.7	10.5	8.0
	£55,000 k+	68.8	47.0	29.4	18.7	12.9	10.2	68.6	46.2	28.3	17.5	11.9	9.2
Family time at home	<£10,000 k	4.2	6.4	7.8	8.2	8.1	7.8	4.2	6.6	8.1	8.6	8.5	8.2
	£10,000–19,999 k	6.6	9.5	10.9	11.1	10.7	10.1	6.5	9.5	10.9	11.0	10.5	9.8
	£20,000–34,999 k	10.6	14.8	16.5	16.3	15.3	14.2	10.1	14.0	15.2	14.7	13.5	12.3
	£35,000–54,999 k	11.2	16.0	18.0	17.8	16.6	15.3	10.5	14.6	16.0	15.3	14.0	12.6
	£55,000 k+	11.1	16.2	18.6	18.7	17.7	16.5	10.5	15.1	17.0	16.7	15.4	14.0

*Note*: Red colour indicates higher probability, green colour lower probability of engaging in a drinking practice.

**TABLE 3:** On‐trade heatmap: for each given age‐sex‐income group, the predicted proportion of their on‐trade occasions that are performances of each practice.

Income ↓		Men						Women					
Age →		20	30	40	50	60	70	20	30	40	50	60	70
On‐trade practices		%	%	%	%	%	%	%	%	%	%	%	%
Male friends at the pub	<£10,000 k	10	13.9	20.6	29.1	35.6	38.9	0	0	0.1	0.1	0.1	0.1
	£10,000–19,999 k	8.8	12.1	17.2	23.1	27.6	30.0	0	0	0.1	0.1	0.1	0.1
	£20,000–34,999 k	11.5	15.6	21.2	27	30.8	32.4	0	0	0.1	0.1	0.1	0.1
	£35,000–54,999 k	8.6	11.8	16.7	22.1	25.8	27.0	0	0	0	0.1	0.1	0.1
	£55,000 k+	6.5	9.0	13.3	19	23.0	24.5	0	0	0	0.1	0.1	0.1
Extended occasion	<£10,000 k	38.5	40.0	28.4	12.4	3.0	0.4	27.1	33.3	25.9	11.8	2.8	0.4
	£10,000–19,999 k	31.2	32.1	21.9	9.1	2.2	0.3	21.0	25.0	18.1	7.6	1.7	0.2
	£20,000–34,999 k	25.5	25.8	16.9	6.6	1.5	0.2	15.3	18.0	12.3	4.8	1.0	0.1
	£35,000–54,999 k	30.7	31.7	21.5	8.8	2.0	0.3	17.6	20.6	14.3	5.6	1.2	0.1
	£55,000 k+	41.3	42.5	30.4	13.4	3.2	0.4	25	28.7	20.4	8.5	1.9	0.2
Family meal	<£10,000 k	3.0	3.3	4.1	5.0	5.4	5.5	8.4	11.1	14.9	19.0	20.6	18.8
	£10,000–19,999 k	4.6	5.0	6.0	6.9	7.4	7.4	12.4	15.8	19.7	23.1	23.7	21.7
	£20,000–34,999 k	5.4	5.9	6.6	7.3	7.5	7.3	13.0	16.4	19.5	21.3	20.7	18.1
	£35,000–54,999 k	5.7	6.3	7.4	8.5	8.9	8.6	13.2	16.5	19.8	21.9	21.4	18.7
	£55,000 k+	5.8	6.4	8.0	9.8	10.7	10.5	14.2	17.5	21.6	24.9	24.9	22.0
Big night out	<£10,000 k	15.5	7.3	4.1	2.4	1.4	0.8	28.0	15.6	9.5	6.0	3.4	1.8
	£10,000–19,999 k	15.4	7.2	3.9	2.2	1.2	0.7	26.6	14.3	8.1	4.7	2.5	1.3
	£20,000–34,999 k	14.4	6.6	3.4	1.8	1.0	0.6	22.2	11.8	6.4	3.4	1.8	0.9
	£35,000–54,999 k	15.1	7.1	3.8	2.1	1.2	0.6	22.2	11.7	6.4	3.5	1.8	0.9
	£55,000 k+	12.9	6.0	3.4	2.0	1.2	0.7	20.0	10.3	5.8	3.3	1.7	0.9
Meeting friends at the pub	<£10,000 k	14.4	14.7	16.9	19.5	20.5	20.3	13.5	16.3	20.4	24.6	25.6	22.9
	£10,000–19,999 k	16.6	16.8	18.4	20.2	20.7	20.4	14.9	17.4	20.2	22.3	22.1	19.7
	£20,000–34,999 k	15.9	15.9	16.7	17.3	17.0	16.2	12.7	14.7	16.3	16.8	15.7	13.4
	£35,000–54,999 k	16.0	16.3	17.8	19.2	19.2	18.3	12.2	14.0	15.7	16.4	15.4	13.1
	£55,000 k+	12.1	12.2	14.1	16.3	17.1	16.4	9.7	10.9	12.6	13.7	13.2	11.4
Quiet drink at the pub	<£10,000 k	13.9	16.7	21.2	25.3	25.7	23.0	3.2	4.6	6.4	8.0	8.0	6.5
	£10,000–19,999 k	17.3	20.7	25.1	28.5	28.2	25.0	3.9	5.3	6.9	7.8	7.5	6.0
	£20,000–34,999 k	16.4	19.4	22.6	24.2	23	19.7	3.3	4.5	5.5	5.8	5.3	4.0
	£35,000–54,999 k	12.9	15.5	18.7	20.9	20.2	17.3	2.4	3.3	4.1	4.4	4.0	3.1
	£55,000 k+	12.0	14.4	18.4	22.1	22.3	19.4	2.4	3.2	4.1	4.6	4.3	3.3
Going out with the partner	<£10,000 k	1.0	1.4	2.1	3.0	3.9	4.8	2.3	3.6	5.9	9.0	11.6	12.7
	£10,000–19,999 k	2.7	3.6	5.2	7.2	9.2	11.0	5.8	8.9	13.4	18.7	23.0	25.2
	£20,000–34,999 k	4.9	6.4	8.6	11.4	13.9	16.2	9.2	13.9	19.8	25.9	30.1	31.4
	£35,000–54,999 k	5.2	7.0	9.9	13.5	16.8	19.5	9.4	14.2	20.5	27.2	31.7	33.1
	£55,000 k+	4.4	5.9	8.7	12.8	16.7	19.7	8.4	12.4	18.4	25.4	30.5	32.1
Meal with friends	<£10,000 k	3.6	2.7	2.7	3.3	4.3	6.3	17.4	15.5	17.0	21.5	27.8	36.9
	£10,000–19,999 k	3.3	2.5	2.4	2.8	3.5	5.1	15.4	13.3	13.6	15.7	19.3	25.6
	£20,000–34,999 k	5.9	4.3	4.0	4.4	5.3	7.5	24.3	20.7	20.2	21.8	25.3	32.0
	£35,000–54,999 k	5.8	4.4	4.2	4.8	5.9	8.3	23.0	19.5	19.2	20.9	24.4	30.9
	£55,000 k+	4.9	3.7	3.7	4.5	5.9	8.4	20.4	16.9	17.1	19.5	23.4	29.9

*Note*: Red colour indicates higher probability, green colour lower probability of engaging in a drinking practice.

### 2.6 Constraints on publishing

The data provider, Kantar, played no role in the research process, including conception, design, analysis, interpretation, write‐up or the decision to publish. Use of this data is allowed under the terms of the contract and nondisclosure agreement between Kantar and the Universities of Sheffield and Glasgow, which requires research outputs to be submitted to the data provider ahead of publication. The data providers' right to request changes is limited to matters of accuracy regarding their data.

## 3 RESULTS

### 3.1 Age, sex and income variations in off‐trade drinking practices

Overall, there appeared to be no clear patterns of sex differences in the level of engagement in the different home‐drinking practices. In contrast, most off‐trade practices showed a strong age gradient. Notably, off‐trade get togethers accounted for 60–70% of young adults' but only 10–20% of older adults' occasions, regardless of sex or level of income (see Figure 1, Table 2). Conversely, evening at home with partner and quiet drink at home were both more common at older ages, while there was no clear age gradient for family time at home.

For two practices, there were also obvious income patterns. For quiet drink at home, which commonly involved drinking alone, the income gap was small among those in their 20s, but at older ages the practice was much more common among those with household incomes below GBP £20,000 than those with higher incomes. In contrast, Evening at home with partner accounted for large occasion shares for high‐income drinkers, at older ages, but was much less common in low‐income drinkers.

### 3.2 Age, sex and income variations in on‐trade drinking practices

Table 3 and Figure 2 show results for on‐trade drinking practices. Male friends at the pub was an almost exclusively male practice, which was less common in younger men but popular among older men, particularly those on lower incomes. Quiet drink at the pub also appeared male‐dominated, with all ages engaging in the practice but again more common in older, lower‐income groups. In contrast, the mixed‐sex group practice Meeting friends at the pub was shared among women and men of all ages. In older women but not men, it appears to account for greater occasion shares among those in lower‐income households.

Three practices accounted for greater shares of women's occasions compared to men's: Family meal, Going out with the partner and Meal with friends. Family meals accounted for around a quarter of female on‐trade occasions in the over‐40s, and somewhat less at younger ages, with no clear income patterning. Going out with the partner similarly accounted for greater shares at older ages but appeared strongly socially stratified, accounting for much smaller shares among those in the bottom two income bands, while income gaps appeared to widen with age. Meal with friends accounted for a large proportion of women's on‐trade occasions at all ages, from around 20% of occasions at age 20 to around 30% in older women. There was no clear income gradient.

Extended occasions, characterised by longer durations, multiple venues, heavy drinking, food and socialising, accounted for the highest share of occasions for younger adults in their 20s and 30s, for smaller shares in midlife and hardly featured among the over‐60s. The income stratification is different to other practices: the practice accounted for around 40% of both the most and least affluent young men's on‐trade occasions but was less common in middle‐income groups. Like extended occasions, Big nights out, again characterised by heavy on‐trade drinking in nightclubs and similar settings, appeared to be mainly a young person's drinking practice and was rare among the over 50s. Extended occasions represented a greater share of young men's than young women's occasions, with the opposite pattern seen for big nights out.

## 4 DISCUSSION

Our results indicate that a country's drinking practices are not equally distributed across society, extending previous typological analyses of national drinking occasion data. In the on‐licensed trade, there appeared to be large distinctions between men and women, with women's practices focusing on lighter, meal‐based drinking and drinking with a partner, particularly at older ages. Conversely, men engaged in more “traditional” British pub drinking, particularly among lower income groups. In the off‐trade, where the majority of British drinking occurs, both sexes engaged in the same practices. However, home‐based drinking practices appeared bound up with age and household composition, and for higher income groups, this typically meant drinking with one's partner. Longer, socialising‐focused drinking with friends, both inside and outside the home, was common among younger age groups across income bands.

Previous Finnish research found that solo home drinking accounts for a greater share of men's alcohol consumption [4]. This is consistent with our results. In our study, quiet drink at home, often alone, accounted for over half of older, low‐income men's drinking occasions. Among those with higher incomes, drinking with a partner/family became increasingly prominent with age, while socialising with friends generally decreased with age possibly due to changing household composition and routines. This contrasts with qualitative work suggesting older people frame their drinking as social experiences [30].

Two decades or so ago, there was regular media outrage about so‐called ladette culture—young women drinking during nights out and getting intoxicated in formerly male‐dominated spaces [31]. While levels of public concern have subsided, our findings highlight a continued importance of heavier, more prolonged drinking practices in young people. These were more common in younger adults, but big nights out and extended occasions together also account for over a fifth of occasions up to age 40 and more than a tenth up to age 50. Similar patterns are also seen in Finland [4, 32] and have been identified in UK qualitative work on how middle‐aged participants in the night‐time economy seek to maintain youthful identities [33].

Home drinking remains under‐explored with regard to socioeconomic differences. Our findings may reflect relationships between household income and composition that our analyses could not control for. However, there is also the potential persistence of more segregated male and female recreation in older working class households, and the greater role of family meals and entertaining the extended family in middle‐class households [34].

In the on‐licensed trade, the greatest income differences relate to meal‐based practices, suggesting a potential affordability barrier limiting lower income households from participating in this relatively expensive form of leisure. The concentration of such practices among more affluent women may also speak to Schmidt's ‘wet feminism’ concept, whereby society grants privileged women the rights to drink and get drunk, whereas low‐income women may be held to different, more restrictive standards [35].

### 4.1 Implications

While there is a long tradition of cross‐country comparative research on national drinking cultures, there has been less focus on cultural phenomena below national level [6, 36] despite some focus on smaller scale ‘social worlds’ of drinking [37]. Our findings suggest that the concept of a single, uniform national drinking culture may not be helpful. We show substantial variations in how and where different societal groups drink. Nevertheless, it is clear that patterns do exist that transcend situations or occasions, where different types of drinking events are scripted enough to be recognisable as practices through the type of persons present, their activities and the location of events in time/space. A national drinking culture might therefore be conceived as the recognition by a substantial part of the population of the norms applying to different contexts where alcoholic beverages are available. This is consistent with the idea that ‘culture’ is a heterogeneous set of cues which call for particular behaviours and discourage others [38, 39]. That is, a national drinking culture exists only because many individuals share knowledge and, given prevalent affordances (provision of beverages, arrangements of settings, legitimised opportunities) that are themselves reinforced by continued engagement in drinking practices, make similar choices.

In practical terms, a more nuanced understanding of the many different roles that alcohol consumption plays in people's lives may be a useful starting point for prevention research. For example, it is unclear if moderation attempts could be more successful if they specifically target subgroups' main drinking practices. On the one hand, these practices are likely to be responsible for a large proportion of consumption. On the other hand, these practices may be deeply ingrained with identities and social circles and therefore the most difficult to alter, so other practices may be an easier starting point. For example, research could test whether it easier to disrupt everyday domestic routines or occasional very heavy drinking with strong bonding/friendship connotations and whether the ease of such disruptions varies across population subgroups. There may be implications for harm reduction research, for example, whether drinking practices contribute to subgroups' differential risk of various alcohol‐attributable harms over and above alcohol consumption volume and frequency [40]. There are some tentative implications for policy or intervention formation. For example, we show that many occasions that are particularly likely to be heavy drinking occasions involve socialising with friends and extended family. Attractive non‐alcoholic substitutes that allow these practices to continue could potentially drive down consumption. Another example concerns more ‘hidden’ consumption, with couples drinking at home in the evening, the most prevalent home drinking occasion. Such occasions are commonly reported by middle‐aged and older affluent couples and over a quarter of such occasions are heavy drinking occasions. Policy‐makers and alcohol charities could whether their planned policies, campaigns or product labelling are likely to work for this consumer group.

### 4.2 Strengths and limitations

This study benefits from a large, uniquely detailed market research dataset of 34,988 drinking occasions reported by 14,742 respondents in 2019. Such data permitted latent class analysis estimation of both on‐trade and off‐trade drinking practices. This provides novel insights into how different social groups drink, both inside and outside the home, as previous event‐level studies have often focused on the readily observable drinking in licensed venues [39]. However, there are two major limitations arising from the data not being collected for academic research purposes. First, it uses non‐probability sampling. While we weight the survey to the UK Census, some selection bias likely remains [41]. However, when we compared reported consumption in Alcovision with British general population surveys and ‘gold‐standard’ sales data, we found Alcovision captures more of the total beverage‐specific per capita sales, and we know that general population surveys struggle with falling response rates and truly representative samples are elusive [42]. The second limitation is that Alcovision does not include some variables that future academic‐led surveys might want to include, for example, ethnicity, health status or negative motivations for drinking. Moreover, the drinking diaries do not ask respondents to identify mixed‐trade occasions. We created these artificially using information on occasion sequencing/timings [7, 8], but found they represented just 10% of occasions which prevented further breakdown to investigate age‐sex‐income variation. Just under 9% of respondents did not report income and were excluded. Sensitivity analyses show that inclusion of such individuals in the models do not materially affect the results and the distribution of occupational grade (a mandatory response variable assessing social class) between those with and without income data was similar, making bias less likely. There was a smaller number of occasions reported by older female drinkers in the two extreme income groups (*n* = 284 for <£10,000, and *n* = 200 for >£55,000, see Table S1) compared to other sex/age/income groups and patterns involving these two groups should be interpreted cautiously. Finally, we do not know the number of persons in the household, so could not adjust household income to disentangle separate associations of affluence and being partnered on drinking habits.

## 5 CONCLUSIONS

We highlight variations in how and where different societal groups (defined by age, sex and income) drink. Nevertheless, it is clear that patterns do exist that transcend situations or occasions, where different types of drinking events are recognisable as practices through the type of persons who are present, their activities and the location of events in time and space.

## AUTHOR CONTRIBUTIONS

Each author certifies that their contribution to this work meets the standards of the International Committee of Medical Journal Editors.

## CONFLICT OF INTEREST STATEMENT

The authors have no interests to declare.

## Supporting information


**Table S1.** Number of occasions by age, sex and income group.^a^
**Table S2a.** Multinomial logit regression of off‐trade occasion types on age, sex, income.
**Table S2b.** Multinomial logit regression of on‐trade occasion types on age, sex, income.

## Data Availability

Data subject to third party restrictions, and subject to a non‐disclosure agreement between the Universities of Sheffield and University of Glasgow, and the data provider Kantar. Access can however be granted for independent verification purposes.
