# Supplementary material for: Drinking practices: The variation of drinking events across intersections of sex, age and household income
Source: Drug Alcohol Rev. 2024 Nov 13;44(1):144–56. doi: 10.1111/dar.13975 (PMC11743237; doi:10.1111/dar.13975)
Supplement: Supplementary file 1 — Table S1. Number of occasions by age, sex and income group.a Table S2a. Multinomial logit regression of off‐trade occasion types on age, sex, income. Table S2b. Multinomial logit regression of on‐trade occasion types on age, sex, income. [file DAR-44-144-s001.docx]

**ONLINE APPENDIX**

**Table S1. Number of occasions by age, sex and income group^a^**

| Age, years | Income (in £1000 p.a.) | Men | Women |
| --- | --- | --- | --- |
| 18-34 | <10 | 642 | 1211 |
|  | 10-20 | 939 | 1515 |
|  | 20-35 | 1294 | 2026 |
|  | 35-55 | 1107 | 1822 |
|  | >55 | 1192 | 1099 |
| 35-54 | <10 | 603 | 355 |
|  | 10-20 | 1177 | 838 |
|  | 20-35 | 1972 | 1281 |
|  | 35-55 | 1848 | 1128 |
|  | >55 | 1472 | 655 |
| 55+ | <10 | 498 | 284 |
|  | 10-20 | 2068 | 802 |
|  | 20-35 | 2753 | 971 |
|  | 35-55 | 1772 | 533 |
|  | >55 | 909 | 200 |

^a^ in models, age in years is entered as a continuous variable, grouped here for producing this sample size table.

**Table S2a – Multinomial logit regression of off-trade occasion types on age, sex, income**

| Practice | Off-trade get together | | | Evening at home with partner | | | Quiet drink at home | | |
| --- | --- | --- | --- | --- | --- | --- | --- | --- | --- |
|  | OR | S.E. | p | OR | S.E. | p | OR | S.E. | p |
| Female | 0.404 | 0.085 | 0.000 | -0.241 | 0.093 | 0.010 | 0.053 | 0.081 | 0.509 |
| Age (divided by 10) | 0.406 | 0.161 | 0.012 | 0.224 | 0.173 | 0.196 | -1.126 | 0.153 | 0.000 |
| Age squared | -0.017 | 0.016 | 0.295 | -0.014 | 0.018 | 0.439 | 0.074 | 0.016 | 0.000 |
| Income (£10-20K) | 0.253 | 0.209 | 0.228 | -0.446 | 0.182 | 0.014 | -0.505 | 0.182 | 0.005 |
| Income (£20-35K) | 0.381 | 0.195 | 0.050 | -1.231 | 0.169 | 0.000 | -1.023 | 0.169 | 0.000 |
| Income (£35-55K) | 0.472 | 0.200 | 0.018 | -1.650 | 0.177 | 0.000 | -1.043 | 0.174 | 0.000 |
| Income (£55K+) | 0.265 | 0.204 | 0.193 | -1.561 | 0.184 | 0.000 | -1.019 | 0.174 | 0.000 |

Notes: Baseline category for the dependent variable: Family time at home. The reference category for household income group is <£10k

**Table S2b – Multinomial logit regression of on-trade occasion types on age, sex, income**

| Practice | Male friends at the pub | | | Extended occasion | | | Family meal | | | Big night out | | | Meeting friends at the pub | | | Quiet drink at the pub | | | Going out with the partner | | |
| --- | --- | --- | --- | --- | --- | --- | --- | --- | --- | --- | --- | --- | --- | --- | --- | --- | --- | --- | --- | --- | --- |
|  | OR | S.E. | p | OR | S.E. | p | OR | S.E. | p | OR | S.E. | p | OR | S.E. | p | OR | S.E. | p | OR | S.E. | p |
| Female | 0.001 | 0.003 | 0.000 | 0.146 | 0.024 | 0.000 | 0.586 | 0.100 | 0.000 | 0.373 | 0.070 | 0.000 | 0.194 | 0.033 | 0.000 | 0.048 | 0.009 | 0.000 | 0.457 | 0.078 | 0.000 |
| Age (divided by 10) | 3.238 | 0.865 | 0.010 | 7.314 | 2.755 | 0.022 | 2.377 | 0.652 | 0.035 | 0.828 | 0.259 | 0.507 | 2.092 | 0.487 | 0.025 | 2.936 | 0.757 | 0.011 | 2.875 | 0.680 | 0.006 |
| Age squared | 0.893 | 0.024 | 0.000 | 0.716 | 0.038 | 0.000 | 0.910 | 0.028 | 0.001 | 0.944 | 0.036 | 0.122 | 0.917 | 0.023 | 0.000 | 0.886 | 0.024 | 0.000 | 0.908 | 0.023 | 0.000 |
| Income (10-20K) | 0.951 | 0.385 | 0.900 | 0.875 | 0.261 | 0.632 | 1.661 | 0.555 | 0.234 | 1.072 | 0.327 | 0.826 | 1.242 | 0.384 | 0.530 | 1.346 | 0.423 | 0.414 | 2.861 | 1.038 | 0.073 |
| Income (20-35K) | 0.701 | 0.271 | 0.270 | 0.403 | 0.113 | 0.000 | 1.109 | 0.338 | 0.747 | 0.566 | 0.156 | 0.005 | 0.674 | 0.199 | 0.102 | 0.723 | 0.216 | 0.200 | 2.858 | 1.004 | 0.064 |
| Income (35-55K) | 0.527 | 0.196 | 0.016 | 0.491 | 0.134 | 0.000 | 1.185 | 0.356 | 0.604 | 0.598 | 0.164 | 0.014 | 0.684 | 0.196 | 0.108 | 0.572 | 0.171 | 0.012 | 3.113 | 1.070 | 0.048 |
| Income (55K+) | 0.477 | 0.187 | 0.005 | 0.786 | 0.222 | 0.335 | 1.442 | 0.455 | 0.331 | 0.608 | 0.179 | 0.028 | 0.613 | 0.186 | 0.037 | 0.638 | 0.198 | 0.068 | 3.119 | 1.121 | 0.059 |

Notes: Baseline category for the dependent variable: Meal with friends. The reference category for household income group is <£10k
